# Supplementary material for: Magnitude of Potential Biases in COVID-19 Vaccine Effectiveness Studies due to Differential Healthcare seeking following Home Testing: Implications for Test Negative Design Studies
Source: medRxiv. 2024 Dec 31:2024.12.30.24319700. Preprint. [Version 1] doi: 10.1101/2024.12.30.24319700 (PMC11722453; doi:10.1101/2024.12.30.24319700)
Supplement: Supplement 1 [file NIHPP2024.12.30.24319700v1-supplement-1.pdf]

medRxiv preprint doi: https://doi.org/10.1101/2024.12.30.24319700; this version posted December 31, 2024. The copyright holder for this preprint (which was not certified by peer review) is the author/funder, who has granted medRxiv a license to display the preprint in perpetuity. This article is a US Government work. It is not subject to copyright under 17 USC 105 and is also made available for use under a CC0 license.

Supplemental Figure 1: Decision tree depicting factors associated with COVID-19 vaccine effectiveness estimation. Chance nodes are shown by circles, which denote outcomes that may occur by chance at each point in the tree with likelihood of occurrence given by the probability shown under each branch (V_{cov}, RDT_v, RDT_u, HS_v, HS_u, HS_vRDT+, HS_vRDT-, HS_uRDT+, and HS_uRDT-). Terminal nodes (triangles) represent the observed study outcomes of cases and controls based on healthcare provider SARS-CoV-2 test result (positive or negative).

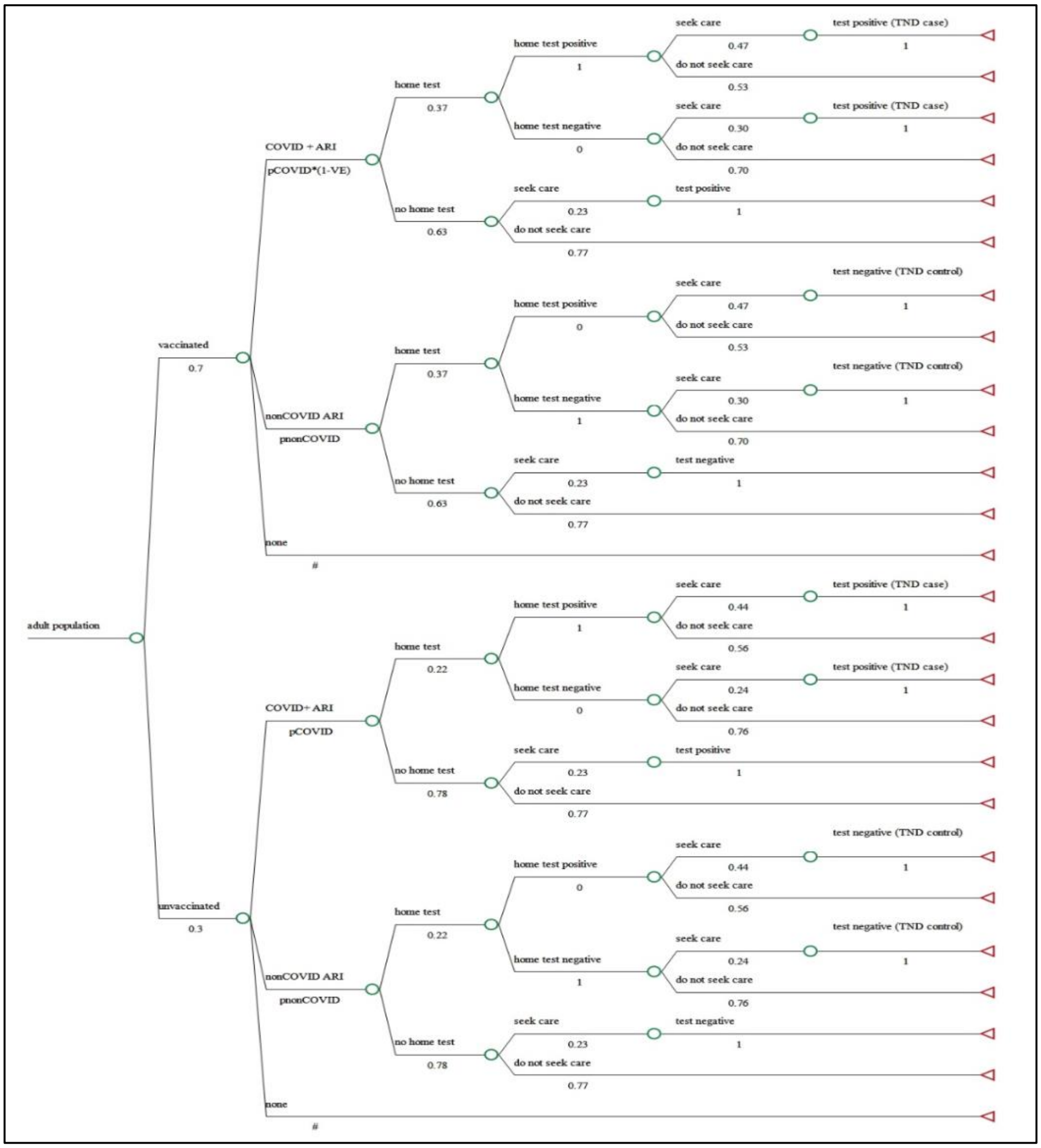

Abbreviations: VE: vaccine effectiveness; V_{cov}: probability of receiving recent COVID-19 booster; RDT_v: probability of home testing among vaccinated; RDT_u: probability of home testing among unvaccinated; HS_v: probability of seeking care among vaccinated given no home testing; HS_u: probability of seeking care among unvaccinated given no home testing; HS_vRDT+: probability of seeking care among vaccinated given positive test; HS_vRDT-: probability of seeking care among vaccinated given negative test; HS_uRDT+: probability of seeking care among unvaccinated given positive test; HS_uRDT-: probability of seeking care among unvaccinated given negative test.

Supplemental Table 1: Beta distribution shape parameters for decision tree factors affecting COVID-19 vaccine effectiveness estimation.

Name	Description	Distribution	Parameter 1: Positive Responses	Parameter 2: Negative Responses	Graph	Minimum	Maximum	Mean	SD	Source
	Vaccination[‡]									
V_{cov}	COVID vaccination uptake	beta	1335	583	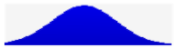	0	1	0.70	0.01	Chasing COVID Cohort
RDT_v	Probability of home testing among vaccinated	beta	497	838	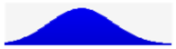	0	1	0.37	0.01	Chasing COVID Cohort
RDT_u	Probability of home testing among unvaccinated	beta	126	457	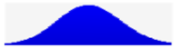	0	1	0.22	0.02	Chasing COVID Cohort
HS_vRDT_+	Probability of seeking care among vaccinated given positive home test	beta	86	98	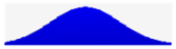	0	1	0.47	0.04	Chasing COVID Cohort
HS_vRDT_-	Probability of seeking care among vaccinated given negative home test	beta	94	219	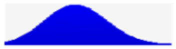	0	1	0.30	0.03	Chasing COVID Cohort
HS_v	Probability of seeking care among vaccinated given no home testing	beta	192	646	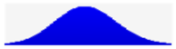	0	1	0.23	0.01	Chasing COVID Cohort
HS_uRDT_+	Probability of seeking care among	beta	28	35	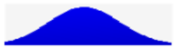	0	1	0.44	0.06	Chasing COVID Cohort

	unvaccinated given positive home test									
HS_uRDT-	Probability of seeking care among unvaccinated given negative home test	beta	15	48	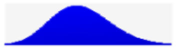	0	1	0.24	0.05	Chasing COVID Cohort
HS_u	Probability of seeking care among unvaccinated given no home testing	beta	105	352	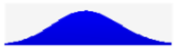	0	1	0.23	0.02	Chasing COVID Cohort

Abbreviations ARI: acute respiratory illness; RDT: rapid diagnostic test; HS: healthcare seeking; SD: standard deviation
Standard deviation for variance of the distribution was calculated using the formula $\sigma = \mu (1-\mu)/(T+1)$; where μ (mean) = $\alpha/\alpha+\beta$; T (precision) = $\alpha+\beta$; and α, β, denote parameters 1 and 2.

medRxiv preprint doi: https://doi.org/10.1101/2024.12.30.24319700; this version posted December 31, 2024. The copyright holder for this preprint (which was not certified by peer review) is the author/funder, who has granted medRxiv a license to display the preprint in perpetuity. This article is a US Government work. It is not subject to copyright under 17 USC 105 and is also made available for use under a CC0 license.

Supplemental Table 2: Definition of simulation parameters from CHASING COVID Cohort study surveys

Parameter notation	Description	Definition	Survey Question
N	Survey population	Study population defined reporting ARI symptoms and have completed the prior survey	Since you completed your last survey, have you had any of the following symptoms? [cough, runny nose, sore throat, shortness of breath]
V_{cov}‡	COVID-19 vaccination uptake	Vaccination status was defined as receiving the booster at or before prior survey fielding date.	Since your last survey, have you received a COVID-19 booster?
RDT	Home testing	Home testing was defined as receiving an at-home RDT and motivated to test due to ARI symptoms	Since you completed your last survey, were any of your viral tests an at-home rapid test?<br><br>and<br><br>If selected yes was tested or tried to get a test, what motivated you to get or try to get a test for COVID-19? Please select all that apply. [I was experiencing COVID-19-like symptoms]
RDT+/-‡‡	SARS-CoV-2 viral test result	SARS-CoV-2 test result among at-home RDT users	If took an at-home rapid test: Since you completed your last survey, what was the result of your at-home rapid test(s)?<br><br>Since you completed your last survey, were any of your viral (PCR or rapid) test(s) positive/reactive?
HS	Healthcare seeking	Healthcare seeking for symptoms	Have you seen or called a physician or health care professional for any of these symptoms?

Abbreviations ARI: acute respiratory illness; RDT: at-home rapid diagnostic test; HS: healthcare seeking

‡ *Vaccination status based on whether respondents reported receiving the most recent booster dose at or before the prior questionnaire's fielding day. For participants who completed the survey questionnaire administered between March and September 2022, vaccination was defined as those who received the second mRNA monovalent COVID-19 booster vaccine (fourth COVID-19 dose). From September 2022 through September 2023, vaccination status was based as those who received the bivalent mRNA COVID-19 vaccine. During October 2023,vaccination was based on those who received the updated 2023-2024 COVID-19 vaccine booster dose.*

medRxiv preprint doi: https://doi.org/10.1101/2024.12.30.24319700; this version posted December 31, 2024. The copyright holder for this preprint (which was not certified by peer review) is the author/funder, who has granted medRxiv a license to display the preprint in perpetuity.
This article is a US Government work. It is not subject to copyright under 17 USC 105 and is also made available for use under a CC0 license.

Unvaccinated participants are those who did not receive the most recent booster dose at or before prior survey's fielding day.

[‡‡] SARS-CoV-2 test result based on at-home RDT use was only ascertained in survey administered in March 2022 (V10); for all other surveys, SARS-CoV-2 viral test result was based on response to any viral test result.

medRxiv preprint doi: https://doi.org/10.1101/2024.12.30.24319700; this version posted December 31, 2024. The copyright holder for this preprint (which was not certified by peer review) is the author/funder, who has granted medRxiv a license to display the preprint in perpetuity. This article is a US Government work. It is not subject to copyright under 17 USC 105 and is also made available for use under a CC0 license.

Supplemental Table 3: Bias between true *VE* and $\widehat{VE}$ based on 10%-point increase or decrease of probability of healthcare seeking among unvaccinated test-negative controls in the base model.

True VE (%)	Probability of healthcare seeking among unvaccinated controls (*HS_uRDT-*)			
	**+ 10%-point *HS_uRDT-***	**Percent bias‡**	**- 10%-point *HS_uRDT-***	**Percent bias‡**
**5**	-8.7	-13.7	9.9	4.9
**10**	-3.0	-13.0	14.6	4.6
**20**	8.5	-11.5	24.1	4.1
**40**	31.3	-8.7	43.1	3.1
**60**	54.2	-5.8	62.1	2.1
**80**	77.1	-2.9	81.0	1.0
**95**	94.28	-0.7	95.26	0.3

‡*Percent bias is the absolute difference between observed VE ($\widehat{VE}$) and true VE.*

Abbreviations ARI: acute respiratory illness; RDT: rapid diagnostic test; HS: healthcare seeking; VE: vaccine COVID-19 effectiveness
